# An Update on the Pathology and Molecular Features of Hodgkin Lymphoma

**DOI:** 10.3390/cancers14112647

**Published:** 2022-05-26

**Authors:** Akira Satou, Taishi Takahara, Shigeo Nakamura

**Affiliations:** 1 Department of Surgical Pathology, Aichi Medical University Hospital, Nagakute 480-1195, Japan; ttakahara@aichi-med-u.ac.jp; 2 Department of Pathology and Laboratory Medicine, Nagoya University Hospital, Nagoya 466-8550, Japan; snakamur@med.nagoya-u.ac.jp

**Keywords:** classic Hodgkin lymphoma, nodular lymphocyte-predominant Hodgkin lymphoma, immune evasion, PD-L1, pathology, molecular feature

## Abstract

**Simple Summary:**

Hodgkin lymphomas (HLs) include two main types, classic HL (CHL) and nodular lymphocyte predominant HL (NLPHL). Recent molecular findings in HLs have contributed to dramatic changes in the treatment and identification of tumor characteristics. For example, PD-1/PD-L1 blockade and brentuximab vedotin, an anti-CD30 antibody bearing a cytotoxic compound, are now widely used in patients with CHL. Biological continuity between NLPHL and T-cell/histiocyte-rich large B-cell lymphoma has been highlighted. An era of novel therapeutics for HL has begun. The aim of this paper is to review the morphologic, immunophenotypic, and molecular features of CHL and NLPHL, which must be understood for the development of novel therapeutics.

**Abstract:**

Hodgkin lymphomas (HLs) are lymphoid neoplasms derived from B cells and consist histologically of large neoplastic cells known as Hodgkin and Reed–Sternberg cells and abundant reactive bystander cells. HLs include two main types, classic HL (CHL) and nodular lymphocyte predominant HL (NLPHL). Recent molecular analyses have revealed that an immune evasion mechanism, particularly the PD-1/PD-L1 pathway, plays a key role in the development of CHL. Other highlighted key pathways in CHL are NF-κB and JAK/STAT. These advances have dramatically changed the treatment for CHL, particularly relapsed/refractory CHL. For example, PD-1 inhibitors are now widely used in relapsed/refractory CHL. Compared with CHL, NLPHL is more characterized by preserved B cell features. Overlapping morphological and molecular features between NLPHL and T-cell/histiocyte-rich large B-cell lymphoma (THRLBCL) have been reported, and biological continuity between these two entities has been highlighted. Some THRLBCLs are considered to represent progression from NLPHLs. With considerable new understanding becoming available from molecular studies in HLs, therapies and classification of HLs are continually evolving. This paper offers a summary of and update on the pathological and molecular features of HLs for a better understanding of the diseases.

## 1. Introduction

Hodgkin lymphomas (HLs) are lymphoid neoplasms derived from B cells [1,2,3,4,5,6]. These neoplasms pathologically consist of large neoplastic cells known as Hodgkin and Reed–Sternberg (HRS) cells, on a background containing extensive non-neoplastic immune cells [7,8]. As one of the most frequent lymphomas, HL accounts for about 10% of such cancers. Its incidence is higher in western countries and lower in Asian countries [9,10,11,12]. Of the two major types, classic HL (CHL) and nodular lymphocyte predominant HL (NLPHL), about 90% are CHL [8,12].

The treatments for CHL, particularly relapsed/refractory CHL, have been dramatically changing. Brentuximab vedotin, an anti-CD30 antibody bearing a cytotoxic compound, is now widely used in relapsed/refractory CHL [13,14]. CD30 is a defining marker of CHL and contributes to cell proliferation and survival [15]. Other promising agents are PD-1 inhibitors, which have shown superior efficacy in CHL [16]. Immune escape mechanisms that underlie the development of CHL contribute to the good response to the PD-1/PD-L1 blockade. HRS cells bear *PD-L1* aberrations and express PD-L1, which are strongly associated with the immune escape features of CHL [17,18,19]. The tumor microenvironment (TME) of CHL has been thoroughly analyzed, and its contributions to the immune escape mechanism of CHL highlighted [20,21,22,23].

Compared with CHL, NLPHL is more characterized by preserved B cell features. Particularly, NLPHL shares a number of features with T-cell/histiocyte-rich large B-cell lymphoma (THRLBCL). Morphologically, NLPHLs with a diffuse pattern resemble THRLBCL, and in the 2017 World Health Organization classification, NLPHLs with a diffuse T-cell-rich pattern are designated as having a THRLBCL-like pattern [8]. Molecular analysis also has highlighted overlapping features between NLPHL and THRLBCL, further supporting the close relationship between the two diseases.

An era of novel therapeutics for HL has begun. The aim of this paper is to review the morphologic, immunophenotypic, and molecular features of CHL and NLPHL, which must be understood for the development of novel therapeutics.

## 2. Classic HL

### 2.1. Clinical and Pathological Features

**Figure 1:**
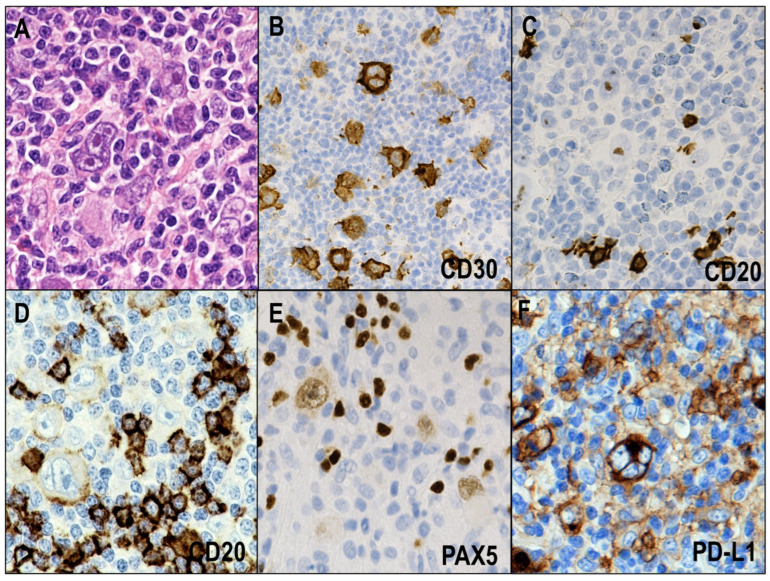
Histological and immunohistochemical features of classic Hodgkin lymphoma (CHL). (**A**) CHL is histologically composed of Hodgkin and Reed–Sternberg (HRS) cells and abundant reactive bystander cells (HE × 400). (**B**) HRS cells are universally positive for CD30 (×400). (**C**,**D**) HRS cells are negative or weakly positive for CD20 (×400). (**E**) HRS cells typically show weak expression of PAX5 (×400). (**F**) HRS cells are highly associated with PD-L1 expression (×400).

CHL is divided into four histological subtypes: nodular sclerosis (NS), mixed cellularity (MC), lymphocyte-rich (LR), and lymphocyte-depleted (LD) [8]. CHL consists histologically of HRS cells and abundant reactive bystander cells, including histiocytes, small lymphocytes, plasma cells, and eosinophils (Figure 1A). HRS cells are universally positive for CD30 (Figure 1B) and variably positive for CD15. These cells show a reduced expression of B-cell markers (Figure 1C,D) and typically show a weak expression of PAX5 (Figure 1E). The association with the Epstein–Barr virus (EBV) varies across the subtypes and is stronger in MCCHL and LDCHL and weaker in NSCHL and LRCHL [24]. An aberrant expression of T cells (e.g., CD4 and CD2) and cytotoxic molecules (TIA1 and granzyme B) has been documented in a small subset (approximately 7%) of the CHL cases with a reference to their poorer prognosis [25,26].

#### 2.1.1. NSCHL

**Figure 2:**
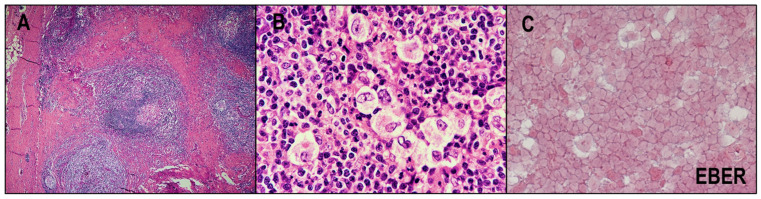
Nodular sclerosing classic Hodgkin lymphoma (NSCHL). (**A**) NSCHL is characterized by thickened lymph node capsules and nodules surrounded by collagen bands (HE × 40). (**B**) HRS cells in NSCHL have abundant clear cytoplasm and are called lacunar cells (HE × 400). (**C**) The EBV-positive rate of NSCHL is the lowest among CHLs. The present case is EBV-negative (×400).

NSCHL is the most frequent of the four subtypes and accounts for 70–80% of CHLs in western countries [8]. The frequency varies across geographic regions, and NSCHL is more common in developed compared with less developed countries [27,28]. This subtype mainly affects adolescents, with frequent mediastinal mass [29,30]. Bulky mass occurs in about half of patients with NSCHL [31]. Histologically, NSCHL is characterized by a thickened lymph node capsule and nodules surrounded by collagen bands (Figure 2A). Nodules contain HRS cells and extensive non-neoplastic immune cells. HRS cells in NSCHL that have abundant clear cytoplasm because of shrinking artifacts are called lacunar cells (Figure 2B). The EBV-positive rate of NSCHL is approximately 10−25%, which is the lowest among CHLs (Figure 2C) [32,33,34]. Generally, the prognosis with NSCHL is better than with other types of CHLs [35].

#### 2.1.2. MCCHL

**Figure 3:**
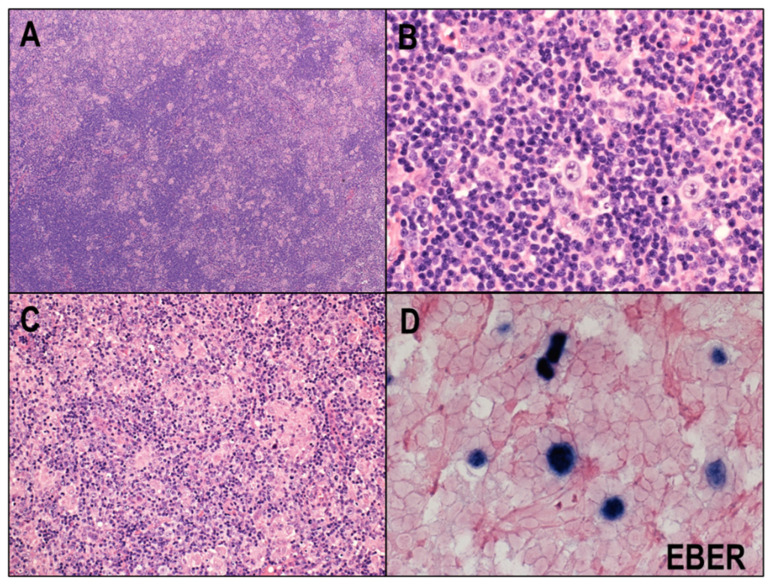
Mixed cellularity classic Hodgkin lymphoma (MCCHL). (**A**) MCCHL lacks a thickened lymph node capsule and broad bands of fibrosis (HE × 40). (**B**) HRS cells in MCCHL show a classic appearance (HE × 400). (**C**) The background cells may contain abundant epithelioid histiocytes, and epithelioid granulomas can be observed (HE × 100). (**D**) MCCHL is highly associated with EBV (×400).

MCCHL is the second most frequent subtype and accounts for 20−25% of CHLs in western countries [8]. This type is more common in older people and immunocompromised individuals, such as those living with HIV [36]. The frequency of this subtype is higher in developing countries, where a childhood peak in incidence is observed [37]. The architecture of the affected lymph node is usually effaced, although some cases may show an interfollicular pattern. In contrast to NSCHL, MCCHL lacks a thickened lymph node capsule and broad bands of fibrosis, and the HRS cells in MCCHL have a classic appearance (Figure 3A,B). The background cells may contain abundant epithelioid histiocytes, and epithelioid granulomas can be observed, particularly in EBV-positive cases (Figure 3C). MCCHL is highly associated with EBV, and the EBV-positive rate with this subtype is around 75% (Figure 3D). The prognosis used to be worse than for NSCHL and better than for LDCHL [38], but these differences have largely disappeared since the advent of modern therapy.

#### 2.1.3. LRCHL

**Figure 4:**
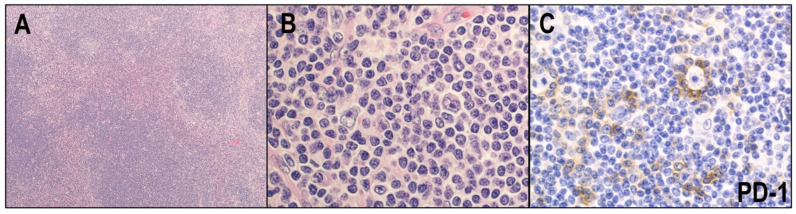
Lymphocyte-rich classic Hodgkin lymphoma (LRCHL). (**A**) LRCHL commonly shows a nodular growth pattern, which may contain germinal centers (HE × 40). (**B**) The nodules are composed of HRS cells and small lymphocytes without polymorphic cell composition (HE × 400). (**C**) HRS cells are frequently characterized by rosetting of PD1-positive T cells (×400).

LRCHL is a rare subtype and accounts for approximately 5% of CHLs. This type occurs in adults, and most patients present in the early stages [39]. Peripheral lymph nodes are typically affected. LRCHL commonly shows a nodular growth pattern, which may contain germinal centers (Figure 4A). The nodules are composed of HRS cells and small lymphocytes without polymorphic cell composition (Figure 4B). HRS cells are frequently characterized by the rosetting of PD1-positive T cells (Figure 4C). Compared with other CHL subtypes, these neoplastic cells often express B-cell transcription factors, such as BOB1 and OCT2, and CD20. Approximately 30–50% of cases are EBV-positive [38]. Morphologically, LRCHL shows overlapping features with NLPHL, and an immunohistochemical examination is essential to differentiate the two. The prognosis with LRCHL is similar to that of NLPHL and slightly better than for the other types of CHL [39,40].

#### 2.1.4. LDCHL

**Figure 5:**
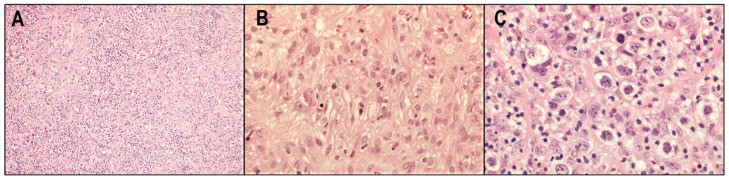
Lymphocyte-depleted classic Hodgkin lymphoma (LDCHL). (**A**) LDCHLs have relatively abundant neoplastic cells and diminished background cells (HE × 40). (**B**) LDCHLs with diffuse fibrosis (HE × 400). (**C**) LDCHLs with rich neoplastic cells showing a pleomorphic appearance (HE × 400).

LDCHL is the rarest subtype and accounts for <2% of CHLs. This type is more frequent in developing countries and in people living with HIV [41,42]. Most patients with LDCHL present with advanced-stage disease and B symptoms [42,43]. Histological features of LDCHLs are relatively abundant neoplastic cells and diminished background cells (Figure 5A). Two patterns are recognized, one of diffuse fibrosis and the other with rich neoplastic cells showing a pleomorphic appearance (Figure 5B,C). Immunostaining is useful to differentiate this type from other lymphomas that show overlapping morphologic features, such as EBV-positive diffuse large B-cell lymphoma or anaplastic large cell lymphoma. LDCHL is highly associated with EBV, and its EBV-positive rate is around 75%. This type is associated with a worse prognosis compared with other types of CHL [44].

### 2.2. Key Pathways and Genetic Lesions in HRS Cells

CHL is frequently characterized by chromosomal abnormalities. Conventional cytogenic studies have revealed that numerical and structural chromosomal aberrations are common in HRS cells [45]. Comparative genomic hybridization studies have revealed that gains and losses also are common [46].

NF-κB is constitutively activated in HRS cells and considered essential for HRS cell survival [47,48]. Previous studies have revealed that the NF-κB components of canonical and non-canonical signaling (e.g., REL, RELB, and p52) play a key role in HRS cell growth and survival [49,50,51,52]. Other studies have highlighted mutations in members of the NF-κB pathway, showing that two mechanisms mediate NF-κB activation. One involves gains and amplifications in *NIK*, *REL*, and *BCL3*, encoding positive regulators of NF-κB [53,54,55]. The other involves inactive mutations (point mutations and deletions) in *TNFAIP3*, *NFKBIE*, *NFKBIA*, *TRAF3*, and *CYLD*, which encode negative regulators of NF-κB [56,57,58,59,60,61]. These findings suggest that both the canonical and non-canonical NF-κB signaling pathways are activated in CHLs. The NF-κB pathway of HRS cells is also activated by a high-level expression of the tumor necrosis factor (TNF) receptor family proteins, such as CD30, CD40, CD95, and RANK. The binding of these receptors by their respective ligands activates downstream signaling pathways, enhancing NF-κB signaling [48,55]. Mast cells and eosinophils that express the CD30 ligand and T cells that express the CD40 ligand often colocalize with HRS cells.

JAK/STAT is another key pathway in CHL. STAT3, STAT5, and STAT6 are constitutively activated and highly expressed in these lymphomas [62,63]. Activation of this pathway promotes HRS cell proliferation and survival. Indeed, in CHL cell lines, the inhibition of JAK2 significantly decreases JAK/STAT signaling and growth. Some studies have identified recurrent genetic lesions that activate the JAK/STAT pathway in CHL. *JAK2* is affected by frequent gain and rare translocation mutations [46,54,64,65]. Recurrent mutations in *STAT6* have been found in about one third of CHLs, whereas the frequency of *STAT3* and *STAT5* mutations is lower [66,67]. Inactivating mutations in *SOCS1* and *PTPN1*, two negative main regulators of the JAK/STAT pathway, have also been identified [68,69]. A recent study using whole-exome sequencing highlighted that the JAK/STAT pathway is genetically dysregulated in 87% of CHL cases [66]. JAK/STAT activation via cytokine signaling also contributes to the survival and proliferation of HRS cells. HRS cells secrete a number of interleukins (e.g., IL-5, 6, 7, 13, 15, and 21) and express receptors of these interleukins [70,71,72,73]. These findings imply a role for an autocrine signaling model of cytokine signaling and JAK/STAT in HRS cells.

AP-1 is a transcription factor composed of proteins belonging to c-Jun, c-Fos, and ATF family members. JUN, JUNB, and ATF3 are highly upregulated in HRS cells, and AP-1 is constitutively activated [74]. Two recent studies highlighted the pathogenic role of the AP-1 family member basic leucine zipper transcription factor, ATF-like 3 (BATF3) [75]. The BATF3 expression mediated by the JAK/STAT pathway is essential for the survival of HRS cells and promotes MYC activity. In a subset of CHLs, S1PR1 expression is increased and drives a feedforward signaling loop to regulate BATF3 [76]. Another constitutively activated pathway in HRS cells is the NOTCH1 pathway. NOTCH1 is highly expressed by HRS cells, and in CHL cell lines, NOTCH1 signaling promotes the survival and proliferation of HRS cells [77].

### 2.3. Immune Evasion Mechanisms in CHL

As described above, HRS cells are characterized by a high somatic mutation load. In addition, in EBV-positive cases, HRS cells express viral proteins, so that these cells theoretically should be a good target for cytotoxic T cells and NK cells, which prevent tumor growth [78,79]. However, CHLs rely on a significant immune evasion mechanism, and HRS cells escape from attack by cytotoxic T cells and NK cells. This immune evasion mechanism plays a key role in the development of CHLs, and CHL is now considered as a representative immune evasion-type lymphoma [80]. Multiple factors contribute to the immune evasion mechanism in HRS cells, and the PD-1/PD-L1 pathway is considered the dominant factor.

PD-L1 is an inhibitory immune checkpoint molecule that suppresses the adaptive arm of the immune system [81,82]. It promotes tumorigenesis by attenuating the activity of tumor-specific CD8^+^ T cells by neutralizing PD-1 expressed on their surface. Two general mechanisms, innate and adaptive immune resistance, drive the PD-L1 expression by cancer cells [81,82]. In innate resistance, constitutive PD-L1 expression is driven by *PD-L1* alterations or aberrant signaling pathways, such as the AKT and STAT3 pathways, which are frequently activated in many cancers. The genetic alterations in *PD-L1* include 9p24.1 gain in lymphomas and gastric adenocarcinomas, *PD-L1* 3’-UTR disruption in multiple cancers, and *CIITA-PD-L1* fusion, which is commonly detected in mediastinal large B-cell lymphoma [18,83,84,85,86,87,88,89,90,91,92,93]. In the adaptive immune resistance, PD-L1 expression by tumor cells is induced by interactions with cytokines in the TME, in particular IFN-γ secreted by activated CD8^+^ T cells, activated Th1-type CD4^+^ T cells, and NK cells. PD-L1 expression by cancer cells is induced through an adaptive response to escape from attack by these immune cells [94].

HRS cells in CHLs are highly associated with PD-L1 expression (Figure 1F). Indeed, we recently reported that around 90% of NSCHLs and MCCHLs express PD-L1 on HRS cells [19]. Some previous studies have highlighted genetic alterations that lead to PD-L1 expression on HRS cells. The most frequent genetic alteration in CHL is 9p24.1 copy gain, and *PD-L1* and *PD-L2* have been identified as key targets of this copy gain [17]. Furthermore, a correlation has been reported between PD-L1 expression and the *PD-L1* copy number in HRS cells. *JAK2* also is included in the broader 9p24.1 amplification region, and *JAK2* copy gain induces JAK2 expression and enhances the JAK/STAT pathway, leading to PD-L1 expression. FISH studies in formalin-fixed paraffin-embedded biopsy specimens of CHLs showed that 99% (107/108) of evaluated cases had *PD-L1* and *PD-L2* aberrations [18]. The aberrations were polysomy (*n* = 5), copy gain (*n* = 61), amplification (*n* = 39), and translocation (*n* = 2).

PD-L1 immunohistochemistry is now considered a useful diagnostic tool for CHL. As mentioned above, most of NSCHLs and MCCHLs express PD-L1 on HRS cells, while HRS-like cells in reactive lymph nodes or neoplastic cells in NLPHL and THRLBCL do not express PD-L1 [19,95,96]. Therefore, PD-L1 immunohistochemistry is helpful to differentiate CHL from reactive lymphoid lesion with HRS-like cells, NLPHL, and THRLBCL. Particularly, it is helpful when the biopsy tissue is small. Immunoblasts in the inflammatory background of angioimmunoblastic T-cell lymphoma (AITL) and nodal peripheral T-cell lymphoma with a TFH phenotype (nPTCL-TFH) are non-neoplastic B cells, and they occasionally mimic HRS cells [97]. AITLs with HRS-like cells, particularly those with a low burden of neoplastic cells, may easily be misdiagnosed as CHL. These HRS-like cells cannot be differentiated from true HRS cells by standard immunostaining used for cHL diagnosis. A recent study suggested that PD-L1 immunohistochemistry is also useful to differentiate these two; PD-L1 expression is highly frequent in HRS cells and rare in HRS-like cells [19].

PD-L1 also is expressed on immune cells in the TME of CHLs. Based on findings using multiple immunofluorescence and digital image analysis, the majority of PD-L1^+^ cells in the TME of CHLs are tumor-associated macrophages (TAMs) [22]. Moreover, these PD-L1^+^ TAMs localize in proximity to HRS cells, suggesting that high-level expression of PD-L1 on TAMs is driven by the response to local cytokine production by HRS cells. Both PD-L1^+^ HRS cells and TAMs bind to PD-1^+^ CD4^+^ and PD-1^+^ CD8^+^ T cells, which contribute to the immune evasion mechanism of CHLs through attenuating the activity of these T cells. Therefore, the total amount of PD-L1 in the vicinity of the HRS cells is increased, which may result in the enhancement of the immune evasion of CHLs. Another study suggested that PD-L1 on TAMs may be directly transferred from HRS cells via a membrane transfer mechanisn known as “trogocytosis” [98].

Other than *PD-L1* aberration, some other genetic lesions associated with immune evasion have been reported. *β2M* is frequently mutated in CHLs and reduces antigen presentation by HRS cells through the downregulation of MHC class I expression. The result is that the recognition of HRS cells by CD8^+^ T cells is impaired [99]. Gene fusions involving *CIITA* have been detected in a certain number of CHLs. *CIITA* is an MHC class II transactivator, and *CIITA* aberrations lead to the downregulation of the MHC class II expression and overexpression of PD-L1 and PD-L2 [90].

In addition to PD-L1^+^ immune cells in TME, other features and interactions between HRS cells and the microenvironment contribute to the immune evasion mechanism of CHLs [78,79]. HRS cells secrete soluble factors with immunosuppressive effects, such as TGFβ, IL-10, and galectin-1. HRS cells also recruit regulatory T cells (Tregs) and myeloid-derived suppressor cells (MDSCs) into the TME. Tregs inhibit tumor-specific T cells by secreting TGFβ and IL-10 and by expressing PD-1, CTLA-4, and PD-L1 on the cell surface. MDSCs strongly regulate the functions of T cells, macrophages, dendritic cells, and NK cells. IDO expression by macrophages and dendritic cells also inhibits T cell and NK cell function. A recent study using single-cell transcriptome analysis identified a novel LAG3^+^ T-cell population in the TME of CHLs. LAG3^+^ T cells have features similar to Tregs and contribute to immune evasion [20].

### 2.4. EBV and CHL

EBV infects more than 90% of the worldwide adult population, and the infection persists for life [100]. EBV preferentially infects B cells through binding to CD21 and human leukocytic antigen on the surface of B lymphocytes. The life cycle of EBV has two phases, lytic replication and latency [101]. In immunocompetent individuals, immune surveillance by EBV-specific cytotoxic T cells regulates proliferation of EBV-infected cells and keeps them in a resting state. In individuals with an immunodeficient status, the number of EBV-specific cytotoxic T cells declines, which can result in the reactivation and proliferation of EBV-infected cells [102]. EBV is associated with some lymphoma, including Burkitt lymphoma, extranodal NK/T cell lymphoma, immunodeficiency-associated lymphoproliferative disorder, EBV-positive diffuse large B-cell lymphoma, and a significant portion of CHLs [8,103]. The frequency of EBV positivity varies in different geographic regions by age and among the subtypes of CHL. In developing countries, the EBV positivity rate is much higher than developed countries. In some developing countries, the rate is almost 100% [27,104]. In developed countries, the CHLs of childhood and old people are commonly EBV-positive and the MC type, whereas the CHLs of young adults are generally EBV-negative and the NS type [105]. In developed countries, a positive rate for each subtype is as follows: MC type (75%), LD type (75%), LR type (30–50%), and NS type (10–25%) [24]. Overall, the EBV positivity rate is about 40% in developed CHLs. Based on the expression pattern of viral proteins, three EBV latency patterns have been recognized. In the latency III (growth program) pattern, all viral latent genes, EBV nuclear antigens (EBNAs 1, 2, and 3A–C), and latent membrane proteins (LMP1, 2A, and 2B) are expressed. In latency II (default program), EBNA1, LMP1, and LMP2A are expressed. Latency I (latency program) is characterized by the restricted expression of EBNA1. The EBV-positive HRS cells show a latency II expression pattern, and EBV-encoded small RNA is expressed in all three patterns [106,107].

The HRS cells in CHL are now thought to originate from germinal center B (GCB) cells [4,5,108]. In addition, all of the HRS cells in EBV-positive CHL are infected by EBV, which indicates that the infection is an early event in the pathogenesis of these neoplastic cells. Therefore, it is assumed that EBV first infects naïve B cells, which in turn enter lymphoid follicles and differentiate into memory B cells through germinal center differentiation. Further differentiation of EBV-infected B cells is blocked by EBNA1, LMP1, and LMP2A, which are constitutively expressed in EBV-positive CHLs. These blocked cells accumulate mutations and transform into neoplastic cells [100,108,109]. Among the three viral latent genes expressed in the default program, EBNA1 is expressed in latency I to III and essential for the replication of the viral genome in cell division. The other two proteins, LMP1 and LMP2A, may play an important role in the tumorigenesis of EBV-positive CHLs. LMP1 mimics CD40, which is a member of the TNF-receptor superfamily and a key receptor of GCB cells. Both LMP1 and CD40 stimulate the NF-κB, PI3K/AKT, and JAK/STAT signaling pathways and rescue B cells from apoptosis and promote their proliferation [110,111]. LMP2A has a function similar to the B-cell receptor (BCR) with the absence of antigen. LMP2A delivers a non-proliferative or tonic signal that is essential for the survival of B cells [112]. As CD40 and BCR signaling are key to GCB cell survival, LMP1 and LMP2A may be important in the survival of EBV-infected GCB cells with destructive *IgV* mutations that can make them a precursor of HRS cells. Indeed, it has been proposed that EBV can rescue crippled GCB cells from apoptosis, and acute EBV infection or infectious mononucleosis is associated with an increased risk for CHL [113,114,115,116,117]. Furthermore, *TNFAIP3* and *NFKBIA* mutations, which activate the NF-κB pathway, are detected mostly in EBV-negative CHLs, indicating that LMP1 plays a vital role in activating NF-κB in EBV-positive CHL [57,118]. Thus, EBV is considered a major pathogenic factor in EBV-positive CHL.

## 3. NLPHL

### 3.1. Clinical and Pathological Features

NLPHL predominantly occurs in males and in young adults. Cervical, axillary, or inguinal lymph nodes are typically affected. Approximately 80% of patients with NLPHLs have stage I/II disease, and the remaining 20% present with advanced-stage disease. These advanced-stage patients may also have bulky mass, hepatosplenomegaly, and/or B symptoms. Up to 20–30% of patients with NLPHL experience progression or recurrence during their disease course. NLPHL generally carries a good prognosis with a 10-year overall survival of >80% [8,119].

**Figure 6:**
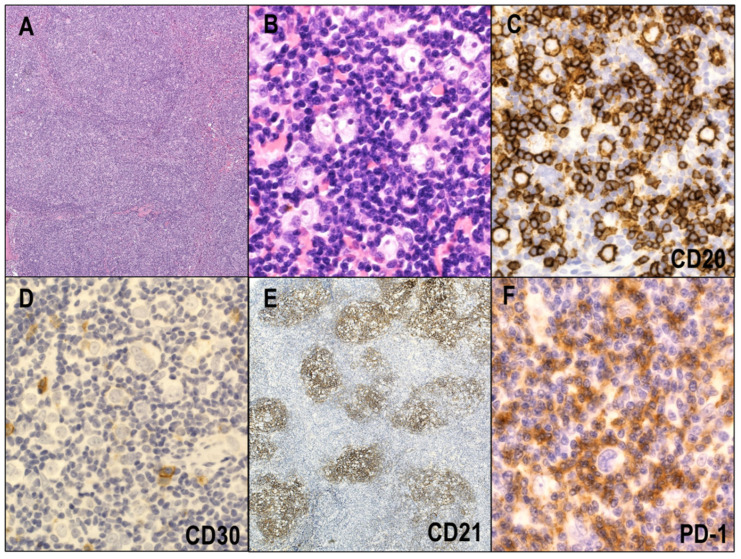
Nodular lymphocyte predominant Hodgkin lymphoma (NLPHL). (**A**) A majority of NLPHL cases are characterized by a nodular growth pattern (HE × 40). (**B**) The nodules consist of abundant small reactive B lymphocytes, epithelioid histiocytes, and intermingled neoplastic cells called lymphocyte-predominant (LP) cells (HE × 400). (**C**) LP cells express CD20 (×400). (**D**) CD30 is rarely expressed in LP cells (×400). (**E**) Immunostaining for CD21 highlights the FDC meshwork within nodules (×40). (**F**) LP cells are characterized by rosetting of PD1-positive T cells (×400).

Histologically, the lymph node involved by NLPHL shows a nodular, nodular and diffuse, or predominantly diffuse pattern. Six histologic patterns have been identified in NLPHL: (A) classical B-cell-rich nodular, (B) serpiginous/interconnected nodular, (C) prominent extranodular LP cells, (D) T-cell-rich nodular, (E) diffuse (THRLBCL-like), and (F) diffuse moth-eaten (B-cell-rich) [8,119]. Most NLPHL cases are characterized by a nodular growth pattern consisting of abundant small reactive B lymphocytes, epithelioid histiocytes, and intermingled neoplastic cells called lymphocyte-predominant (LP) cells, which reside within and outside nodules (Figure 6A,B). The nodules are characterized by a meshwork of follicular dendritic cells (FDCs). These typical histological findings are detected in patterns A and B, whereas small reactive T lymphocytes dominate nodules or diffuse infiltrate of patterns C to F, and the FDC meshwork is diminished in the diffuse area. The morphological findings of the diffuse area resemble those of THRBCL. Thus, NLPHL cases with a dominant diffuse area are difficult to distinguish from THRBCL. Of note, patients with patterns C to F are more likely to present with advanced-stage disease and bone marrow involvement compared with those who have pattern A or B [120,121,122]. Prominent sclerosis is occasionally found, particularly with recurrence [123].

Immunohistochemically, LP cells show a strong overlap with GCB cells and express pan B-cell markers, such as CD20, CD19, and CD79a (Figure 6C). GCB cell markers (BCL6, HGAL, and LMO2) are expressed on LP cells with the exception of CD10. Therefore, CD10 is the only marker that may be useful for distinguishing LP cells from GCB cells. Unlike HRS cells in CHL, LP cells strongly express the B-cell transcription factors PAX5, OCT2, and BOB1. CD30 and CD15 are rarely expressed on LP cells (Figure 6D), a trait that also can be used to distinguish between HRS cells and LP cells. Immunostaining for CD21 highlights the FDC meshwork within nodules of NLPHL (Figure 6E). LP cells are characterized by the rosetting of PD1-positive T cells (Figure 6F), and the rosette formation is commonly observed around LP cells in the nodular area and less frequently in the diffuse area of NLPHL. EBV is only rarely detected in LP cells [124,125].

Patients with NLPHL typically experience an indolent clinical course. However, a subset of patients suffers multiple recurrences and progression to large B-cell lymphoma. The progression occurs in up to 30% of NLPHL cases, and large B-cell lymphoma components show histologic features of THRLBCL or DLBCL [126,127,128,129].

### 3.2. Key Pathways and Genetic Lesions in LP Cells

Little is known about signaling pathways and genetic alterations in NLPHL. Similar to CHL, LP cells show constitutive activation of the JAK/STAT pathway, which is found in around 50% of NLPHL cases. This activation is caused by mutations in SOCS1, a negative regulator of JAK2 [130]. Another pathway that is frequently activated in LP and HRS cells is the NF-κB pathway [131]. However, LP cells rarely harbor *TNFAIP3* and *NFKBIA* mutations, which are frequently detected in HRS cells and activate the NF-κB pathway [132]. Furthermore, LP cells lack evidence of EBV infection, indicating that LMP1 does not contribute to the activation of the NF-κB pathway in LP cells. Therefore, the mechanisms of constitutive activation of the NF-κB pathway seem to be different between LP and HRS cells, and those of LP cells remain to be elucidated. A very recent paper revealed that Moraxella catarrhalis-derived antigens were detected in LP cells of some NLPHLs, which may contribute to the lymphomagenesis through additive activation effects on the BCR and the NF-κB pathways [133]. Frequent *BCL6* translocation and recurrent deletion in a region on 9p11 have been found in LP cells [134,135]. These two abnormalities have not been identified in HRS cells. The alteration of genes targeted by somatic hypermutation, such as *PAX5*, *PIM1*, *RHOH,* and *MYC*, has been identified in 80% of NLPHLs [136]. Highly recurrent mutations in *SGK1*, *DUSP22,* and *JUNB* have also been reported in NLPHLs [137].

### 3.3. Relationship between NLPHL and THRLBCL

In the last two decades, many studies have highlighted the biological continuity between NLPHL and THRLBCL [131,137,138,139]. Based on the pathogenic mechanism, THRLBCL can be divided into two types, de novo and progression from NLPHL.

As noted above, NLPHL and THRLBCL share morphological features, and the morphological findings of a diffuse area resemble those of THRBCL. Thus, NLPHL cases with a dominant diffuse area, particularly the THRLBCL-like/pattern E, are difficult to distinguish from THRBCL. A molecular analysis also has highlighted overlapping features between NLPHL and THRLBCL. Gene expression profiling the analysis of microdissected tumor cells from NLPHL and THRLBCL has revealed strong similarities between the two. An array comparative genomic hybridization analysis of microdissected tumor cells of NLPHL, THRLBCL-like NLPHL, and THRLBCL has shown that two regions on 2p16 and 2p11 are affected in all three [131]. Moreover, the targeted sequencing of NLPHL and THRLBCL has shown that they share recurrent mutations, including those in *JUNB*, *DUSP22*, and *SGK1*, which further supports commonalities between the two [137,139].

## 4. Conclusions

Recent molecular findings in HLs have contributed to dramatic changes in the treatment and identification of tumor characteristics. Many studies have suggested that immune escape mechanisms, particularly involving the PD1/PD-L1 axis, are closely associated with development of CHL, and the PD-1/PD-L1 blockade is now widely used in patients with CHL. Brentuximab vedotin, an anti-CD30 antibody bearing a cytotoxic compound, is also widely used in CHLs. Overlapping morphological and molecular features between NLPHL and THRLBCL have been reported, and biological continuity between these two entities has been highlighted. Some THRLBCLs are considered to represent progression from NLPHLs. Therefore, CHL and NLPHL are considered quite distinct diseases, and NLPHL may be reclassified as B-cell lymphoma in the near future.

With considerable new understanding becoming available from molecular studies in HLs, the therapies and classification of HLs are continually evolving. This paper offers a summary of and update on the pathological and molecular features of HLs for a better understanding of the diseases.
